# Intensity-modulated radiotherapy with simultaneous integrated boost for locoregionally advanced nasopharyngeal carcinoma

**DOI:** 10.1186/1748-717X-9-56

**Published:** 2014-02-18

**Authors:** Junlin Yi, Xiaodong Huang, Li Gao, Jingwei Luo, Shiping Zhang, Kai Wang, Yuan Qu, Jianping Xiao, Guozhen Xu

**Affiliations:** 1 Department of Radiation Oncology, Cancer Hospital, Chinese Academy of Medical Sciences, NO 17, Panjiayuan nanli, Chaoyang District, Beijing 100021, China

**Keywords:** Locally advanced, Nasopharyngeal carcinoma, Concurrent chemoradiotherapy, Intensity-modulated radiotherapy with simultaneous integrated boost

## Abstract

**Objective:**

To compare the treatment outcomes of intensity-modulated radiotherapy with simultaneous integrated boost (IMRT-SIB) alone to concurrent chemoradiotherapy (CCRT) for locoregionally advanced nasopharyngeal carcinoma (NPC).

**Methods:**

From November 2001 to December 2009, 333 patients with pathologically diagnosed, locoregionally advanced NPC were treated by IMRT-SIB with or without weekly cisplatin concurrent chemotherapy at our institute. Among them, 62 patients received neo- or adjuvant chemotherapy or molecular target drugs were excluded from this analysis. There were 129 patients received IMRT-SIB alone, and 142 patients received IMRT-SIB with weekly cisplatin 30 mg/m^2^ for 7 weeks. The radiotherapy protocol was identical for each group.

**Results:**

There were no significant differences in survival between CCRT and IMRT-SIB group in terms of gender, T/N classifications and concurrent chemoradiotherapy. The 5-year local control (LC), overall survival (OS), disease-free survival (DFS) and distant metastasis-free survival (DMFS) for the entire group were 87.0%, 79.4%, 69.7 and 83.3%, respectively. The LC, OS, DFS and DMFS for CCRT and IMRT-SIB alone groups were 80.6% vs. 90.8% (P = 0.10), 71.7% vs. 83.2% (P = 0.201), 63.9% vs. 74.6% (P = 0.07), and 79.6% vs. 86.0% (P = 0.27), respectively.

**Conclusion:**

Compared to CCRT, IMRT-SIB alone had demonstrated similar disease LC, OS, DFS and DMFS in locoregionally advanced NPC. Careful radiation target volume design and simultaneous integrated boost may play a role that overrides the benefit from concurrent chemotherapy. Further investigation with randomized study is necessary to determine whether IMRT-SIB alone can achieve similar outcomes of concurrent chemoradiotherapy.

## Introduction

Majority patients with nasopharyngeal carcinomas (NPC) present with locoregionally advanced stages at diagnosis, the treatment modality with concurrent chemoradiotherapy has demonstrated superior to radiotherapy (RT) alone in terms of disease locoregional control and survivals, cisplatin based chemoradiotherapy has improved 5-year overall survival from 50% to 75% [1-6]. In the past 15-years, IMRT technique has widely utilized in the treatment of head and neck cancers, particularly in NPC due to anatomic complexity and benefits in accurately encompassing tumor targets and reducing organ at risk’s (OAR) toxicities. Two-dimensional (2D) or 3-dimensional techniques (3D) has been used in the previous published NPC studies, it is unknown whether IMRT alone with accurate tumor delineations and the technique of IMRT-SIB could achieve similar outcomes of disease loco-regional control. The theory supporting this hypothesis is the majority of NPC in Asia are poor or undifferentiated histology, and NPC is a type of sensitive tumor to RT or CCRT, comparing 2D conventional RT, sophisticated IMRT-SIB provides the advantage of adequate target coverage with concaved shape of nasopharyngeal carcinoma target volume, and limit toxicity to adjacent critical structures [7,8]. Several studies have shown that compared to 2D technique, IMRT improved NPC disease local controls and reduced radiation associated toxicities [9-11]. It is reasonable to postulate that the local control improvement of nasopharyngeal carcinoma treated by IMRT-SIB will be translated into overall survival benefit. To investigate whether IMRT-SIB alone can achieve outcomes that are as effective as the published data on locoregional control and survival in NPC patients, we conducted a retrospective cohort study for patients with locoregionally advanced NPC that were all treated with IMRT-SIB technique alone or concurrent with weekly cisplatin. The results may provide some clinical evidences that IMRT-SIB may be applied for selected locoregionally advanced NPC.

## Methods

### Patient’s inclusion criteria

From November 2001 through December 2009, totally 416 pathologically proven nasopharyngeal carcinoma patients were treated with IMRT-SIB technique in our hospital. There were 333 patients in stage III, IVA/B when reevaluated according to the 6^th^ UICC staging system, sixty-two patients were excluded from this study for receiving induction and/or adjuvant chemotherapy (n = 20), or epidermal growth factor receptor inhibitors (n = 42). There were 197 males and 74 females; the median age was 47 (range, 12-81). Initial evaluation included complete physical examination, fiberoptic nasopharyngoscopy, chest image (X-ray film and CT scan for N3 patients), MRI or CT of the nasopharynx including skull base and neck region, blood routine tests, and complete metabolic chemistries. Part of patients came from a clinical trial in which the role of concurrent chemoradiotherapy were compared regardless of the treatment technique, by this reason, there were 129 patients received IMRT-SIB alone, and 142 patients received IMRT-SIB with current chemotherapy.

### Radiotherapy

All patients were treated with 6MV-X by Varian 600C/D linear accelerator, 5 fractions per week, with a total treatment time of 6.5 weeks. The radiotherapy protocol for IMRT-SIB alone and CCRT group was identical. The whole neck IMRT technique was used to cover the primary lesion, nodal disease and entire neck including supraclavicular region. The prescription dose to T1 and T2 primary lesion (GTVp) was 70 Gy in 33 fractions at 2.12 Gy per fraction, while 74 Gy at 2.24 Gy per fraction to T3 or T4 disease and involved retropharyngeal nodes with largest diameter >1.5 cm, all positive lymph node (GTVnd) were given 70 Gy at 2.12 Gy per fraction. The elective radiation dose of 60 Gy at 1.82 Gy per fraction encompasses the high risk regions including uninvolved skull base, parapharyngeal space, posterior one-third of nasal cavity and high risk nodal levels. If there were no positive neck node in the neck, 50-54 Gy was delivered to the bilateral lower neck and supraclavicular region using a two-phase IMRT plan, with phase 1 IMRT plan (28 treatment fractions) cover primary lesion, positive nodes, high risk region and the lower neck/supraclavicular region, the phase 2 IMRT plan (5 treatment fractions) cover only the primary lesion, positive nodes and high risk regions. The dose constraints for major organs at risk were shown as follow: brain stem with 3 mm margin, Dmax < 54 Gy; spinal cord with 5 mm margin, Dmax < 40 Gy; optic nerve, chiasm and temporal lobe, Dmax < 54 Gy; parotid gland, V30-35 < 50%.

### Chemotherapy

Planned chemotherapy was consisted of weekly intravenous cisplatin at 30 mg/m^2^ for 7 weeks.

### Management of residual primary lesions

At the end of treatment, there were 53 patients have residual disease at the primary sites that were documented by MRI and/or endoscopic examination: 22 (15.5%) in CCRT group and 31 (24.0%) in IMRT-SIB alone group. Salvage local treatments include IMRT boost with a mean dose of 8 Gy (range, 4-15 Gy) at 2-3 Gy per fraction (n = 25) or Linac-based stereotactic radiotherapy treatment (SRT) 20 Gy (range, 10-24 Gy) at 2-4 Gy per fraction (n = 28).

### Intra- and Post-treatment assessments

Tumor response was assessed at 50 Gy by clinical exam including endoscopy and/or imaging study. If the volume and geometry of primary lesion and positive nodes were changed significantly, the second CT-simulation was performed and PTV volumes were modified corresponding to significant GTVs regression. A new IMRT plan was designed and started at the 29^th^ treatment fraction in order to avoid treatment break. Patients received physical examination once a week during the treatment, the radiotherapy related toxicities were graded according to the Acute and Late Scoring Criteria of the Radiation Therapy Oncology Group and chemotherapy-related toxicities was evaluated by the CTC 2.0 or CTCAE3.0. If there were grade 4 hematology toxicities and radiotherapy-related mucositis occurred, the treatment would suspend until the toxicities were recovered. The first post-treatment follow-up was at 1 month, then every 3 months for the first 2 years, every 6 months thereafter for 3-5 years, and then once a year.

### Statistical methods

The statistic was performed by SPSS 13.0 software, Kaplan-Meier method was used for calculating the survival, and Log-rank test was used for evaluating the differences between the two groups.

## Results

### Patient characteristics

From November 2001 to December 2009, there were totally 271 newly diagnosed locally advanced nasopharyngeal carcinoma treated with IMRT-SIB with or without concurrent cisplatin-based chemotherapy in our institution. The characteristics of patients and treatment modality were shown in Table 1. There were well balanced between the groups of IMRT-SIB alone and CCRT in terms of gender, age, T classification, clinical stage, and median RT dose to the GTVs.

**Table 1:** The characteristics of patients between IMRT-SIB and CCRT

**Items**	**IMRT-SIB**	**CCRT group**	**p**
	**n = 129**	**n = 142**	
	**No of patients (%)**	**No of patients (%)**	
Gender			0.63
Male	92 (71.3)	105 (73.9)	
Female	37 (28.7)	37 (26.1)	
Age (year)			0.45
≤46	65 (50.4)	65 (45.8)	
>46	64 (49.6)	77 (54.2)	
Pathology			0.02
Non-keratinization			
Differentiated	16 (9.3)	33 (23.2)	
Undifferentiatied	113 (90.7)	109 (76.7)	
T stage			0.45
T1	15 (11.6)	15 (10.6)	
T2	27 (20.9)	29 (20.4)	
T3	60 (46.5)	56 (39.4)	
T4	27 (20.9)	42 (29.6)	
Stage			0.18
III	89 (69.0)	87 (61.3)	
IV	40 (31.0)	55 (38.7)	
Median dose to GTV	74	74	0.12

IMRT-SIB: Intensity-modulated Radiotherapy with Simultaneous Integrated Boost, CCRT: Concurrent chemoradiotherapy, GTV: Gross tumor volume.

### Patient compliance

All patients completed RT as planned. Of 142 patients in CCRT group, 127 (89.4%) received 5 and more cycles of weekly chemotherapy, while 15 patients (10.6%) had 4 cycles or less.

### Acute toxicities

Acute toxicities presenting in the groups of CCRT and IMRT-SIB alone were: grade 3 mucositis, 24.1% versus 29.3% (P = 0.531); grade 2 and 3 neutropenia, 32.7% versus 8%, and 2% versus 0%, respectively (P = 0.008); grade 1 anemia 42.8% versus 24.5%, grade 2 anemia, 6% versus 0% (p = 0.066). Grade 3 radiation dermatitis was 11.1% in both CCRT and IMRT-SIB alone group. Only 1 patient in CCRT group had grade 1 abnormal liver function tests. There were no grade3 or higher anemia observed in either group.

### Treatment outcomes

The 5-year LC, OS, DFS and DMFS for the entire group were 87.0%, 79.4%, 69.7 and 83.3%, respectively. The 5-year LC, OS, DFS and DMFS of CCRT versus IMRT-SIB-alone groups were 80.6% and 90.8% (P = 0.10), 71.7% and 83.2% (P = 0.201), 63.9% and 74.6% (P = 0.07), 79.6% and 86.0% (P = 0.27), respectively. For stage IVA patients, the 5-year DFS, DMFS were 34.3% and 74.7% (p = 0.01), 68.1% and 95.5% (p = 0.03) in CCRT (n = 39) and IMRT-SIB alone group (n = 24), respectively. For N0-1 patients, the 5-year DMFS were 67.3% and 96.9% (p = 0.02) in CCRT (n = 32) and IMRT-SIB alone group (n = 35).

**Figure 1:**
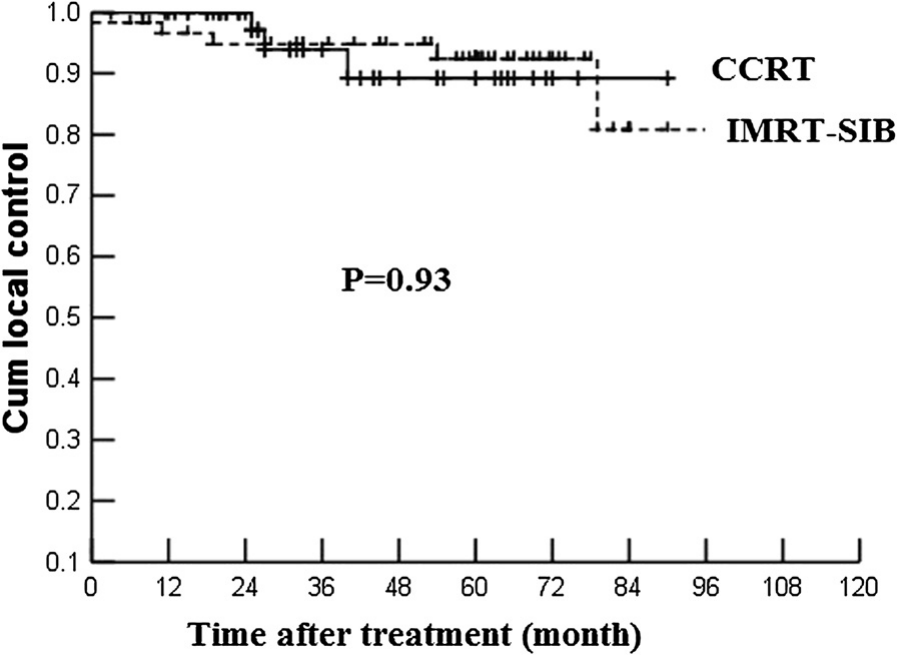
The local control rate of T3 patients treated by IMRT-SIB alone and CCRT.

**Figure 2:**
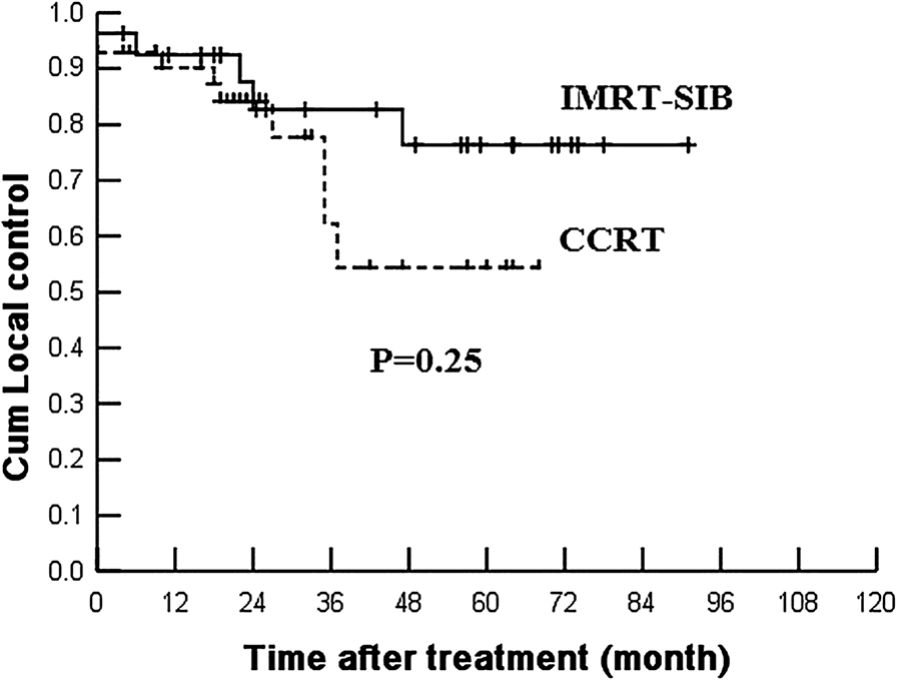
The local control rate of T4 patients treated by IMRT-SIB alone and CCRT.

Univariate analysis including age, gender, T stage and clinical stage showed that there were no significant differences of overall survival between CCRT and IMRT-SIB alone groups (Table 2). No independent prognosis factor was found by multivariate analysis. Figures 1 and 2 showed the local control of T3 and T4 patients.

**Table 2:** The differences of treatment outcomes between CCRT and IMRT-SIB RT alone

**Items**	**LC**		**P**	**OS**		**P**	**DFS**		**P**	**DMFS**		**P**
	**CCRT**	**RT**		**CCRT**	**RT**		**CCRT**	**RT**		**CCRT**	**RT**	
Gender			0.13			0.30			0.1			0.30
Male	77.9	89.9		70.2	77.8		62.9	72.3		79.9	81.4	
Female	93.4	92.9		81.0	89.0		70.0	79.7		81.9	91.3	
Age			0.09			0.23			0.08			0.24
≤46	87.2	94.8		73.7	90.2		68.5	79.7		77.6	90.2	
>46	72.7	86.3		69.2	71.3		57.7	68.3		81.2	80.9	
T3	89.2	92.4	0.93	69.3	84.0	0.45	63.2	68.4	0.31	77.4	81.9	0.82
T4	54.4	76.4	0.25	66.2	67.8	0.59	41.5	64.5	0.15	77.1	91.3	0.08
N0-1	73.4	84.0	0.85	72.2	76.7	0.35	51.5	82.9	0.09	67.3	96.9	0.02
N2-3	81.8	93.5	0.05	71.5	80.4	0.36	65.4	71.6	0.24	81.7	81.8	0.93
III	91.9	95.6	0.35	84.2	88.3	0.94	77.5	80.1	0.57	89.0	86.1	0.77
IV	63.8	63.6	0.352	54.3	71.5	0.24	43.6	61.3	0.15	64.8	86.3	0.12
IVA	47.1	78.6	0.11	62.2	69.2	0.43	34.3	74.7	0.01	68.1	95.5	0.03
IVB	84.0	64.0	0.55	50.0	73.9	0.41	55.6	45.0	0.62	62.5	73.1	0.86
Whole group	80.6	90.8	0.10	71.7	83.2	0.20	63.9	74.6	0.07	79.6	86.0	0.27

LC: Local control; OS: Overall survival; DFS: Disease-free survival; DMFS: Distant metastasis-free survival; RT: IMRT-SIB alone; CCRT: Concurrent chemoradiotherapy.

## Discussion

Several randomized clinical trials have demonstrated that concurrent chemoradiotherapy is superior to radiotherapy alone in the treatment of locoregionally advanced nasopharyngeal carcinoma. Al-sarraf et al. [1] first reported that, compared to RT alone, concurrent cisplatin chemoradiotherapy and adjuvant chemotherapy with cisplatin and 5-Fluorouracil improved 5 years overall survival from 37% to 67% (p = 0.001). Wee et al. [6] confirmed the advantage of CCRT with adjuvant chemotherapy in NPC epidemic regions. At the same period, there were more and more data from nasopharyngeal carcinoma epidemic areas showed that CCRT with or without adjuvant chemotherapy improved the overall survival when compared with RT alone [3,5,12]. These clinical trials confirmed that advantage of CCRT with or without adjuvant chemotherapy have improved local-regional controls [1,5,6], some studies revealed decreases of distant metastases [3,4,12] or both locoregional control and distant metastases [1,5,6]. The meta-analysis also showed that the CCRT was superior to RT alone in nasopharyngeal carcinoma epidemic areas [13]. In Chen’s study [14], 508 patients were randomly assigned to the CCRT followed by adjuvant chemotherapy (n = 251) and CCRT alone group (n = 257), the 2-year overall survival was similar, 94% and 92 (p = 0.32). Based on these evidences, CCRT with or without adjuvant chemotherapy has become the standard care for locoregionally advanced nasopharyngeal carcinoma. However, most of these evidences of standard treatment for locoregionally advanced nasopharyngeal carcinoma were based on the 2D or non-IMRT technique. As the IMRT technique has been widely used in the last decades, and outcome of significant disease locoregional control, people started to reconsider the role of CCRT. In Hong Kong NPC clinical trials (NPC 9901/9902), there were 50% patients received either 3D conformal or IMRT, the results showed that there were no significant differences in terms of overall survival between the groups of CCRT and RT alone [15,16], although the combined analysis of Hong Kong NPC 9901/9902 studies revealed that patients who received CCRT did well in overall survival [17].

Due to the complex anatomic location of nasopharynx, it is technically challenging to deliver the definitive dose to field without partially missing GTV coverage using 2-D RT technique. Such problem can be avoided with the use of IMRT. Furthermore, with IMRT-SIB technique, there are two aspects of radiobiology rationale to improve treatment results: shorten the overall treatment time and increase the fractionation dose to gross tumor volumes [18]. Several studies have confirmed the safety and efficacy of IMRT-SIB in the treatment of NPC. Lee et al. [9], Kwong et al. [19] and Xiao et al. [20] all reported very promising results of locally advanced nasopharyngeal carcinoma treated with IMRT-SIB. In Xiao’s study [20], the prescription dose to NPC GTVs was 68 Gy given in 30 fractions, 2.27 Gy per fraction. The 5-year disease local control, disease-free and overall survivals were 94.9%, 76.7% and 74.5%, respectively. All toxicities were expected, and no exceed treatment complications due to use of IMRT-SIB. In the past 10 years, IMRT-SIB has been more often used in locoregionally advanced head and neck cancer including NPC treatments. Large sample studies from NPC epidemic areas have demonstrated outcome improvements, see Table 3.

**Table 3:** The outcome of NPC treated by 2D technique and IMRT-SIB in epidemic areas

**Author and era**	**No of pts**	**Stage**	**Treatment technique**	**CCRT**	**OS %**	**PFS %**	**DMFS %**
Lee 2005 [21]	2,687	I-IVB	2D	14% CCRT, 9% seq	75	63	81
Yeh 2005 [22]	849	I-IVB	2D	No	59	52	74.7
Yi 200 6 [23]	905	I-IVB	2D	2.8% seq	76.1	58.4	79.8
Lin 2010 [24]	370	IIB-IVB	IMRT	Seq without CCRT	89 (3 yr)	81(3 yr) *	86(3 yr)
Wang 2013 [25]	300	I-IVB	IMRT	Stage III/IV with CCRT	86.1 (4 yr)	NA	85.0
Su 2011 [26]	865	I-IVB	IMRT	222 with CCRT	83.0	NA	84.0
This study	271	III-IVB	IMRT	142 with CCRT	79.4	63.9*	79.6

CCRT: Concurrent chemoradiotherapy; OS: Overall survival; PFS: Progression-free survival.

*: Disease-free survival; Seq: Sequential, 2D: Two dimension; IMRT: Intensity-modulated radiotherapy.

Our results did not demonstrate any significant differences in disease local control, overall survival, disease-free survival and distant-metastasis free survival between patients treated with IMRT-SIB alone or with concurrent chemotherapy. Besides the better dose coverage provided by IMRT technique compared to the 2D technique, there were several other reasons may interpret our results. One is the prescription doses to the gross target volume were 74 Gy with 2.24 Gy/fraction for T3/T4 lesions, the equivalent biological dose were 75.5 Gy if given by 2 Gy/fraction according to the L-Q model, which is about 7.8% increased of total dose when compared to 70 Gy/2 Gy per fraction in the 2D era. If we refer to dose-response curve for head and neck cancer [27], with a γ37 of 2, the local control rate of our patients would be 15.6% higher than those patients who received 70 Gy with 2 Gy/fraction in the 2D era, Actually, the local control rate of RT alone groups in those randomized clinical trials compared the concurrent chemoradiotherapy with radiotherapy alone in 2D era were about 67%-72% [1,5,6], while the 5-year local control of our study was 87%, which was about 15% higher, which may extrapolate a potential increase of overall survival. The reason for choosing a higher prescription dose for T3/4 lesions was based on that there were larger tumor burden in T3/4 lesions than those in T1/2 lesions, and a larger tumor burden needs a higher dose to control. Sze et al. [28] found that the overall correlation between T stage and tumor volume was strongly significant in 308 nasopharyngeal carcinoma patients, the risk of local failure was estimated to increase by 1% for every 1 cm^3^ increase in primary tumor volume. Secondly, with IMRT-SIB, the overall treatment time were 45 days for T3/4 patients, there were 6 days reduced when compared with those who treated by 2D conventional radiotherapy. Shorten overall treatment time will benefit to overcome the tumor cell repopulation and potentially improve disease local control. Also, in our series, there were 53 patients with residual lesions at the end of treatment, received additional boost either by IMRT or SRT. All these factors would improve our treatment outcomes compared to those come from 2D era in the literature. Our previously published data also showed the patients with residual lesions received boost dose irradiation had the same local control as those who had no residual disease at the end of RT [24]. Teo et al. [29] reported dose-escalation above 66 Gy significantly improved local control for T1/T2a and T3/4 tumors when compared to 2D Ho's technique.

Su et al. [26] also found that, comparing to IMRT alone, adding chemotherapy to IMRT did not improve the outcomes for locally advanced NPC. In their study, of 603 patients with T3-4 N2-3 NPC treated with IMRT, 101 were treated with IMRT alone, 222 patients with cisplatin-based CCRT (CCRT group), 207 patients with induction chemotherapy followed by CCRT (IC + CCRT group), 38 patients received IMRT with either induction chemotherapy or adjuvant chemotherapy (IC or Adj group) and 35 had CCRT followed by adjuvant chemotherapy (Con + Adj group). The 5 years overall survival was 77.2% for IMRT group alone, 78.7% for CCRT group, 73.4% for IC + CCRT group, 82.3% for IC or Adj group and 80.6% for Con + Adj group (P = 0.59). There were no significant survival differences no matter them received chemotherapy or not. Of those with locoregionally advanced NPCs who will benefit from CCRT or RT alone? Lin et al. [30] divided the stage III/IVM0 (1992 AJCC staging system) patients from their previously randomized trial [5] into high risk group and lower risk group. The high risk group included patients who met at least one of following criteria: nodal size >6 cm, supraclavicular node metastases, 1992 AJCC stage T4N2, multiple neck node metastases with at least 1 node >4 cm; the others without any high risk factors were in low risk group. The results showed that only low risk group patients benefited from CCRT compared to the RT alone. The 5-year disease LC and OS were 95.1% and 85.3% in CCRT group, the disease local control improvement has been translated into an OS benefit. But, when compared to the Lin’s data, in our study, LC and OS for stage III patients (similar to Lin’s lower risk group) treated with IMRT-SIB alone were 95.6% and 88.3%, is similar to those in Lin’s lower risk group who treated with CCRT. That is to say, in some selected patients (for example, UICC stage III patients), IMRT-SIB alone may be an alternative treatment choice.

Several pitfalls need to be addressed. Selection bias in a retrospective study could have impacted on our results. However, we included all consecutive patients with stage III and non-metastatic stage IV NPC treated with IMRT-SIB in our institution except for those received neo- or adjuvant chemotherapy or molecular target drugs. Second, the cumulative cisplatin dose from our weekly chemotherapy regimen was 180-210 mg/m^2^, which was lower than the recommended standard total dose of 300 mg/m^2^, as there was a positive relationship between the total cisplatin dose and treatment outcomes locally advanced squamous cell carcinoma of head and neck [31]. As CCRT with high acute treatment-related toxicities in patients who received cisplatin regimen of 100 mg/m^2^, about 30% patients could not receive the third cycle of cisplatin [32]. Kim et al. [33] compared and analyzed the tumor response, the overall survival, the toxicity and the chemotherapy dose intensity in the patients with locally advanced nasopharyngeal cancer who were treated with a 3-week cycle of 100 mg/m2 cisplatin or 30 mg/m^2^ weekly cisplatin, the author conclude that weekly 30 mg/m2 cisplatin-based CCRT is a practical, feasible cisplatin schedule for the patients with locally advanced nasopharyngeal cancer in regard to decreasing the interruption of radiation treatment and the treatment-related acute toxicities. So the total dose in our study might be reasonable. We are not clear about the worse outcomes in terms of DFS and DMFS in CCRT group compared to IMRT-SIB alone group for stage IVA and N0-1 patients, the possible reasons maybe the sample size and the bias of a retrospective study.

## Conclusion

IMRT-SIB alone achieved similar treatment outcomes as compared to concurrent chemotherapy and IMRT-SIB in terms of disease local control and overall survival. However, such results need further investigation in a prospective randomized clinical trial.

### Ethical statement

1. This study has been approved by ethics committee of cancer hospital, Chinese academy of Medical Sciences.

2. All patients have given their written consent to be included in research studies prior to their treatment.

## Competing interest

This manuscript was sponsored by National Natural Science Foundation of China (81172125). Junlin Yi, Xiaodong Huang, Li Gao, Jingwei Luo, Shiping Zhang, Kai Wang, Yuan Qu, Jianping Xiao, Guozhen Xu declare no conflict of interest.

## Authors’ contributions

JLY drafted the manuscript, XDH, JWL, SPZ, KW, YQ and JPX participate this study, mainly work for treating patients and data collection. LG and XGZ designed this study. All authors read and approved the final manuscript.
